# What Is the Acheulean?

**DOI:** 10.1002/evan.70029

**Published:** 2026-04-09

**Authors:** Marie‐Helene Moncel, Carolina Cucart‐Mora, Marta Arzarello, Nick Ashton, Javier Baena, Deborah Barsky, Ignacio de la Torre, Nena Galanidou, Paula García‐Medrano, Christine Hertler, Gadi Herzlinger, Thomas Ingicco, Hao Li, Dongdong Ma, Marina Mosquera, Andreu Ollé, Shanti Pappu, Shuwen Pei, Chun Tian, Wei Wang, Christopher J. Bae

**Affiliations:** ^1^ UMR 7194 ‐ CNRS‐ Histoire Naturelle des Humanités préhistoriques Museum National d'Histoire Naturelle, Institut de Paléontologie Humaine Paris France; ^2^ Dipartimento di Studi Umanistici Università degli Studi di Ferrara Ferrara Italy; ^3^ Department Britain, Europe & Prehistory British Museum, Franks House London UK; ^4^ Department of Prehistory and Archaeology Autonomous University of Madrid (Campus Campoblanco) Madrid Spain; ^5^ Institut Català de Paleoecologia Humana i Evolució Social (IPHES‐CERCA) Tarragona Spain; ^6^ Departament d'Història i Història de l'Art Universitat Rovira i Virgili Tarragona Spain; ^7^ Department of Archaeology Institute of History, Consejo Superior de Investigaciones Científicas (CSIC) Madrid Spain; ^8^ Department of History and Archaeology University of Crete Greece; ^9^ Centro Nacional de Investigación Sobre Evolución Humana (CENIEH) Burgos Spain; ^10^ ROCEEH Research Center Senckenberg Research Institute Frankfurt Germany; ^11^ ROCEEH Research Center Heidelberg Academy of Sciences and Humanities Heidelberg Germany; ^12^ School of Archaeology and Maritime Cultures, The Zinman Institute of Archaeology University of Haifa Israel; ^13^ State Key Laboratory of Tibetan Plateau Earth System, Resources and Environment (TPESER) Institute of Tibetan Plateau Research, Chinese Academy of Sciences Beijing China; ^14^ Key Laboratory of Vertebrate Evolution and Human Origins Institute of Vertebrate Paleontology and Paleoanthropology, Chinese Academy of Sciences Beijing China; ^15^ School of Interwovern Science and Arts Krea University Sri City Andhra Pradesh India; ^16^ Sharma Centre for Heritage Education Chennai Tamil Nadu India; ^17^ Institute of Cultural Heritage Shandong University Qingdao People's Republic of China; ^18^ Department of Anthropology University of Hawai'i at Manoa Honolulu HI USA

**Keywords:** Acheulean, early pleistocene, Eurasia, Middle Pleistocene (Chibanian), palaeolithic

## Abstract

The Acheulean represents the longest cultural period known to human history, lasting globally for more than 1.75 million years. It may have emerged as early as 1.95 Ma in Africa, spreading throughout much of the continent and then into Eurasia and lasting up to 350–200 ka in western Europe and South Asia, and even later in eastern Asia. Originally defined in the 1870s, the term Acheulean is one of the earliest and most contested classifications in prehistoric archaeology. Almost 150 years after its first appearance, it remains a source of continuous debate. This paper summarizes roundtable discussions that took place at the *Musée de l'Homme* (Paris) in November 2025 that focused on the meaning of the Acheulean and the diversity of its manifestations across Eurasia. Some 20 researchers, from various institutions across Europe, Asia, and the Pacific participated in this event, during which it became clear that the Acheulean had different meanings to the participants. Among the major points raised during the meeting was the question of how different specialists differentiate the Acheulean from the older Oldowan techno‐complex, with specificities emerging from each of their respective regions of study. The geographic origins and hominin species' attribution of the Acheulean toolmakers were also brought to the fore since important questions have been raised in the last decades by the growing record of hominin taxa that existed during this timeframe across Eurasia and the relatively late arrival of this techno‐complex in Europe. These issues become even more important when we consider the recent evidence emanating out of Asia, which indicates that hominins were present well before their earliest appearance in Europe. The purpose of this paper is not only to make a statement regarding how to define the Acheulean, but also to illustrate its diversity across Eurasia.

## 1 Introduction

**Figure 1:**
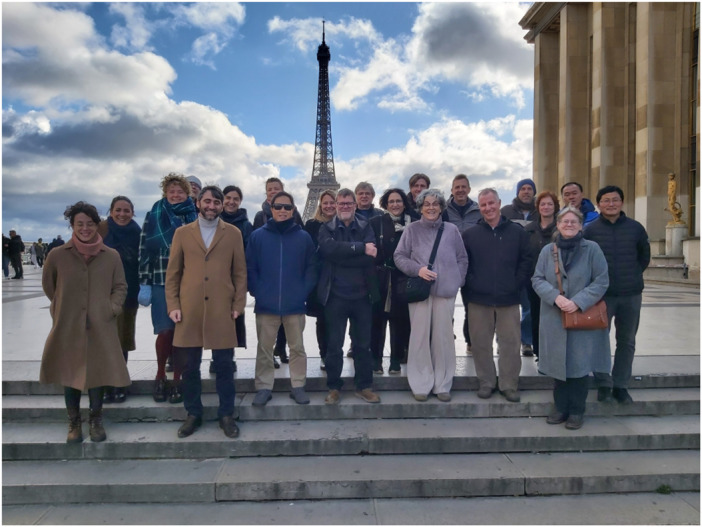
Group photo of the participants from the What is the Acheulean? Conference that was held in the *Musée de l'Homme* in Paris in November, 2025.

In order to delve more deeply into the question of the Acheulean and how it manifests across Eurasia, a workshop was organized in the *Musée de l'Homme* in Paris in November 2025 as part of the European Research Council funded Lateurope project (Directed by M.H. Moncel, ID 101052653), a program whose principal objective is to synthesize and understand the late occupation of western Europe compared to the rest of Eurasia. Given the numerous new discoveries of sites with Acheulean tools across Eurasia and re‐analyses of older lithic collections, we felt it was an opportune time to organize this small conference. Some 20 researchers from different theoretical, methodological, and regional backgrounds participated, traveling from various institutions across Europe and Asia, and even as far away as Hawai'i (Figure 1). The primary goals of the meeting were twofold: (1) to explore how the Acheulean is defined across Eurasia; and (2) to discuss questions related to the hominins responsible for the stone toolkits documented in different areas of the world. Participants also addressed the late manifestations of the Acheulean in Europe relative to Africa and Asia, as well as considering possible dispersal routes and questions relating to convergent evolution. Based on the archaeological evidence presented by the contributors it became clear to us that the Acheulean has divergent meanings to specialists working in different geographical areas and time periods. How the participants define the Acheulean, particularly in the respective regions with which they are most familiar, is presented here to illustrate how diversely it has come to be represented across Eurasia. What follows is a synthesis of the key points of general agreement (not to be confused with universal agreement) by the conference participants.

The term “*Acheuléen*” derives from the site of Saint‐Acheul, a suburb of the city of Amiens in northern France, where this lithic industry was first described in 1872 by Gabriel de Mortillet, a major French prehistorian [1, 2]. This stage is defined by a “fossil directeur,” the biface (handaxe). Mortillet (1872) classified prehistoric periods on the basis of some typical artifacts and defined fourteen cultural phases, using the names of archaeological sites to describe and distinguish them. The “*Acheuléen”* follows the “*Chelléen”* (named after the site of Chelles in eastern France) and is characterized by well‐worked bifaces in what was believed to be a linear technical evolution compared to earlier periods. According to this view, the *Chelléen* was considered an Early Acheulean, marked by more crudely made bifaces. Although Mortillet did not discover the Acheulean, he named, described, and incorporated it into the first scientific cultural framework of prehistory (Acheulean, Mousterian, Solutrean, Magdalenian). Other classification systems have since been proposed, such as the Mode system put forth by Clark [3] and subdivisions within the Oldowan and Acheulean complexes proposed by Leakey [4].

In spite of these attempts to draw up a universal system for the evolution of ancient human culture, the conference participants emphasized that such eponymous site‐based divisions within the Palaeolithic are not necessarily uniformly applicable across Eurasia and Africa. For example, the technological “Stone Age” categories, widely used to classify Palaeolithic assemblages in the African context into three main categories (Early, Middle, Late), is a meaningful way to account for the behavioral variability in Africa compared to western Europe. Similar variability in nomenclatures is noteworthy in South Asia, with differential use of either African or European terminologies for the Palaeolithic. In some cases, regional nomenclatures such as the “Madrasien”, a name for the Acheulean in India [5], have continued to be used until recently, where the dual Lower Palaeolithic cultures of the Acheulean and pre‐Acheulean (Soanian) are now considered the norm (see [6] for further discussion). Another example is the question raised by Gao and Norton [7] (see also [8]) about the utility of simply applying the western European three‐stage Palaeolithic cultural sequence (Lower, Middle, Upper) to the Chinese record. In their view, the Chinese Palaeolithic stone tools can be assigned to one of two different cultural periods (Early and Late) given the absence or paucity of a distinct “Middle Palaeolithic” (see also [9, 10, 11, 12] for broader discussion across eastern Asia). Another point of debate regarding whether the eastern Asian stone tool assemblages can be assigned to the western European Palaeolithic classification system is the presence/absence of the Acheulean east of the so‐called Movius Line [13] (see below).

In order to evaluate variation within the Acheulean tool complex itself, Sharon and Barsky [14] proposed a three‐phase sequence by characterizing the Early Acheulean, the Large Flake Acheulean (LFA) [15] (and references therein) and the Late Acheulean, based on information from key sites situated in the Levant, and then expanding this model to test its validity on a regional level. The LFA is characterized by the development of a diversity of volumetrically complex operative schemes designed to obtain blanks that require little modification to be transformed into standardized morphologies known as LCTs (Large Cutting Tools). According to this view, it was the first Acheulean to arrive in western Europe, where it is mostly present on the Iberian Peninsula and has been reported in rare occurrences in southwestern France and Greece [14, 16, 17, 18, 19, 20]. There was broad agreement that one of the most widely accepted routes this technology diffused from is the Out of Africa expansion through the Levantine corridor [21, 22]. In this context, some of the western European sites fit within the Late Acheulean as defined from eastern Europe [22, 23]. The Anatolian and the Aegean records, namely Kaletepe Deresi 3 and Rodafnidia, that extend the Acheulean map to the north and west of the Levantine corridor, support a more complex, multivariate scenario [24, 25]. Good evidence of this connection may be found in the oldest Acheulean site in Europe, Barranc de la Boella (Spain), that is documented as having analogous features to Early Acheulean sites in Africa and the Near East [23, 26]. Alternatively, technomorphological similarities in Large Flake production between North African and Spanish sites could suggest that these complex technologies may have arrived in southwestern Europe via the Strait of Gibraltar, though this connection may be considered more tenuous [21].

**Figure 2:**
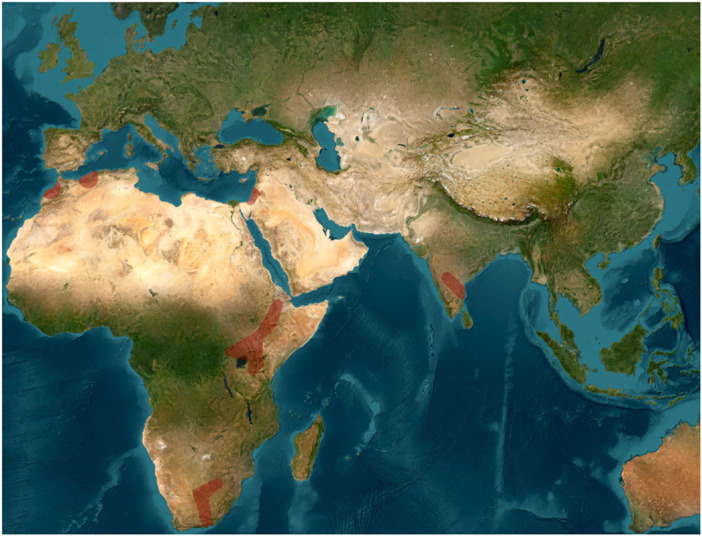
Minimum geographic distribution of early Acheulean biface occurrences (between 1.95 Ma and 1.0 Ma) in Africa and Eurasia. Red shade represents inferred areas of presence derived from clustered, well‐dated archaeological site locations. Envelopes were generated using distance‐based clustering and concave hull reconstruction, followed by moderate spatial expansion.

**Figure 3:**
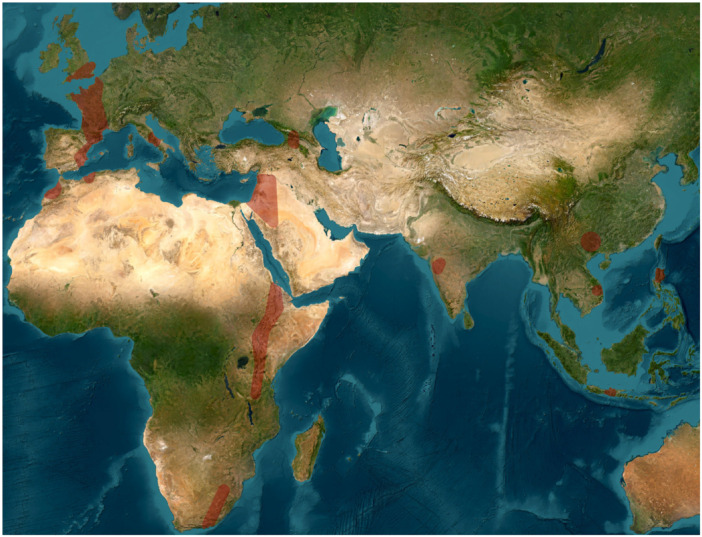
Minimum geographic distribution of Acheulean biface occurrences between 1.0 and 0.5 Ma in Africa and Eurasia. Red shade represents inferred areas of presence derived from clustered, well‐dated archaeological site locations. Envelopes were generated using distance‐based clustering and concave hull reconstruction, followed by moderate spatial expansion.

**Figure 4:**
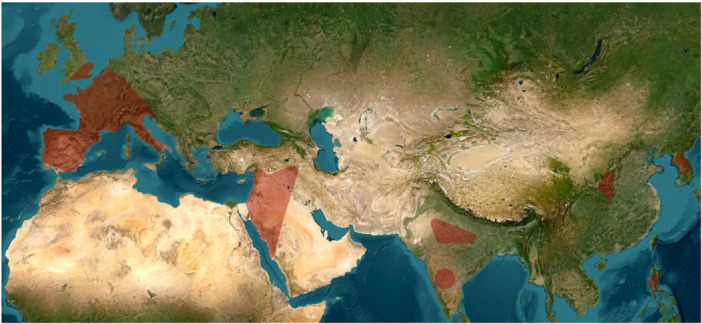
Close‐up on the latest Acheulean biface occurrences with a focus on Eurasia between 0.5 and 0.1 Ma. Minimal geographical distribution is shown by a red shade representing inferred areas of presence derived from clustered, well‐dated archaeological site locations. Envelopes were generated using distance‐based clustering and concave hull reconstruction, followed by moderate spatial expansion.

Participants agreed that these tentative divisions of the Acheulean can be roughly constrained chrono‐spatially as follows, though clearly not applicable across time and space: the **Early Acheulean** (˜from 1.9 to 1.8 Ma) corresponds with the emergence phase found in East Africa with some early “Out of Africa” dispersals into the Levant such as at ‘Ubeidiya, and some later evidence across broader Eurasia (between 1.9 and 1 Ma); the **Large Flake Acheulean** (“LFA”) or “Classical Acheulean” (1.0 Ma–0.5 Ma in Africa and later in different regions of Eurasia) reflects the demographic spread of standardized techno‐behaviors with hominins enlarging their capacity to spread into a range of environments; and the **Late Acheulean** (from 0.5 Ma up to 0.1 Ma or younger), with increased innovation and more frequent inter‐group contacts, as well as sharpened regional distinctions [27]. To synthesize, participants decided to focus on the general distribution of biface localities on well‐dated sites that are presented in Figures 2, 3, 4 and broken up into separate chronological units (Figure 2: 1.9 Ma–1.0 Ma; Figure 3: 1.0 Ma–0.5 Ma; Figure 4: 0.5 Ma–0.1 Ma).

During the workshop, it was admitted that each phase is marked by a set of regionally specific expressions of technological and, very likely, social developments. These distinct phases are represented in temporal succession to one another, although not all of them are present in every region. Further, these sequences are subject to local variation. For instance, in India, an early LFA dates back to 1.5 Ma–1.07 Ma and continues through time, with handaxes and cleavers noted from early levels. The Late Acheulean in contrast, is poorly defined and dated in India. Many of the Acheulean stone toolkits in eastern Asia are younger and would fall into the Late Acheulean chronologically, despite displaying little‐to‐no evidence of increased innovation. Based on morphological grounds, many of these eastern Asian handaxes would probably be classified as Early Acheulean or LFA [28, 29, 30, 31]. At the heart of Eurasia, the LFA is widespread on Lesvos, sharing industrial features with Gesher Benot Ya'aqov (GBY), the iconic LFA assemblage of the Levant. However, the Lesvos sites all appear to be redeposited in horizons dated to the second half of the Middle Pleistocene, and only minimum ages are available for the stone tools [18, 20, 32, 33]. Should these Aegean assemblages then be treated as LFA or Late Acheulean?

Much of this discussion about whether or not this or that stone tool assemblage can be assigned to this or that Palaeolithic (or in Africa, Stone Age) subdivision assumes *a priori* that archaeologists are in agreement regarding the definition of each of these classificatory systems. For instance, are the Oldowan or the Acheulean categorizations defined the same way across Eurasia and Africa? Here, we explore further how the Acheulean is defined across Eurasia and Africa. But before we delve into that discussion it is important to discuss who may have actually produced the Acheulean.

## 2 Can We Identify Which Hominin(s) Produced the Acheulean?

There was large agreement from the participants that any assignment of a hominin species to Acheulean stone tool making is bound to remain a working hypothesis, even when hominin fossils are found in contextual association with an Acheulean assemblage. Through analogical reasoning, species that are coterminous with Acheulean assemblages are treated as possible candidates for the Acheulean innovation and production. But from a technological and behavioral point of view, does it really matter if we can identify the ‘genius’? And if so, was it one or many different ones? Given that we are dealing with more than 1.75 million years of geological time, when in all likelihood, a variety of different hominins produced and used Acheulean technologies there is a wide range of hominins that were present across Eurasia and Africa; to name but a few: *Homo ergaster, H. erectus, H. antecessor, H. bodoensis, H. neanderthalensis, H. juluensis, H. longi, H. sapiens*, and geographically restricted taxa like *H. naledi, H. floresiensis*, and *H. luzonensis* may have overlapped somewhat in time with the Acheulean. And this does not even include species like *H. georgicus, H. caucasi, H. heidelbergensis*, and *H. rhodesiensis* (see various discussions regarding how to treat these latter taxa for example, [34, 35, 36, 37]). A good example of a hominin found in association with Acheulean stone tools is the Middle Pleistocene site of Notarchirico in southern Italy. A juvenile femoral shaft fragment was found in 1985 at the site and in a recent analysis of the fossil, it was tentatively assigned to the “controversial *taxon* [*sic*] *Homo heidelbergensis*” [38]: 8. As the authors themselves acknowledge, by using the word “controversial”, *H. heidelbergensis* is currently a strongly debated taxon [38, 39].

It may only be possible to say that a particular hominin produced the Acheulean in those regions where only one hominin taxon has been proposed to have been present during a restricted time interval. A good example, provided by the Chinese participants, is southern China and the earliest handaxes which currently date to ~780 ka in the Bose Basin [12, 30, 40, 41, 42, 43]. Although other hominins (e.g., *H. juluensis, H. longi*) are present in China during the latter part of the Chibanian [12, 35], during the early Chibanian, only *H. erectus* has been proposed to have lived in southern China during this time period. In a case like this, we can say, with a fair degree of confidence, that *H. erectus* at ~780 ka produced the bifacially worked implements in the Bose Basin in southern China. Of course, this argument would need to be modified if it turns out that *H. juluensis* and/or *H. longi* (or another species yet to be identified) were actually present in southern China during the same time period. Although another hominid, *Gigantopithecus*, was clearly present in the region at this time as well, no studies exist to our knowledge that suggest they could have made and used stone tools. Except for cases like these however, participants emphasised that it would probably be more parsimonious to keep the behavior and biology discussions separate until the two records are detailed enough where one can contribute to a better understanding of the other. As it stands, the two records are usually too fragmentary to be able to say that this or that particular hominin taxon produced the Acheulean. The Notachirico case described above is a good example of how complex this picture is.

## 3 Contemporary Views on the Acheulean Technocomplex

There was agreement that the Acheulean is considered one of the most important technological developments of the Early Stone Age and a key threshold in technological, behavioral, and cognitive evolution, particularly given its level of technological standardization [15, 17, 23, 28, 44, 45, 46, 47, 48]. Original definitions of the “Acheulean” indicate that its main defining feature is the capacity to produce large flakes, a break with the previous Oldowan technocomplex, though it is generally accepted that the Acheulean often appears together with Oldowan core and flake industries [49, 50, 51, 52]. Further, the presence of particular typological and technological elements in a chronological regional range that conforms to a set of tools and by‐products like handaxes, cleavers, and trihedral pieces are important variables in the identification of the Acheulean technocomplex. This is equally true of the small retouched toolkits that begin to include converging edge formats and true points.

The Acheulean is often characterized by complex strategies to produce volumetrically and morphologically controlled blanks that were frequently transformed by subsequent modification into large shaped instruments or small retouched tools, resulting in sets of intentionally configured tool types; characteristics that are a marked break with the Oldowan. Some features, as pointed out by some of the participants, such as bifacial symmetry and measures of edge angle and face regularity, are relatively straightforward to identify, and several analytical approaches and tools have been developed to express them [53, 54, 55, 56]. As the standardization and diversification of these tool groups widened, so did the selection of various lithic raw materials that came to be chosen depending on their qualitative features and suitability in relation to tool types, for either the LCTs or the retouched items. Participants broadly consider that standardization and the capacity to apply a fixed set of different technological procedures to produce controlled flakes for the LFA and subsequently configure them into LCTs are important aspects of the techno‐complex on the whole (handaxes, cleavers, picks); notwithstanding other significant features, such as the wide array of small retouched tools that invariably accompany them and behavioral shifts indicating higher investment in complex social comportments. Indeed, not all Acheulean assemblages include a heavy‐duty toolkit and presence/absence could depend on such factors as regional traditions or even function of the sites [57, 58]. In most assemblages, heavy‐duty tools actually account for only a small proportion of the entire collection [59, 60, 61].

In general, participants emphasized the fact that the Acheulean is also characterized by a plethora of retouched tools. Further, there is a diversification in retouch types that, during the Oldowan, show technomorphological overlap with cores on flakes in some areas. Other features, agreed during the conference discussions that are unevenly spread through time and space, contribute to defining the Acheulean: Tool type variability in relation to raw material diversity; selected blank and extended areas of raw material procurement; bone tool production; spatially structured habitats; increased demography; specialized hunting and standardized butchery techniques, and, importantly, fire making [62, 63]. The emergence of bifacial stone knapping is not necessarily a result of the morphology of the original blank. For example, some participants emphasized that regarding bifacial stone knapping, identified at Atapuerca's Gran Dolina site in level TD‐6, dated to around 0.9 Ma, where some flat cobbles, which can serve as a preform for biface shaping, were expressly avoided [64].

Beyond shaped tools, large and small, the technologies used to manage cores are also considered by the participants to be important indicators of the Acheulean *sensu lato*. Large flake production (e.g., for making handaxes in the LFA) usually is limited to technical constraints and applied with a diversity of methods, including unifacial and non‐alternate bifacial methods [15]. Nevertheless, Acheulean stone reduction can be defined by its hierarchical and volumetric management of knapping, bifaciality, recurrence, and even overcoming raw material constraints [65], underlining important implications about hominin behavioral plasticity and technological flexibility. Unifacial and orthogonal production, widespread in Oldowan toolkits, continue to be represented, while extended operative schemes can lead to bifacial centripetal strategies that, in some cases, result in a relatively precocious appearance of Levallois production in western Europe around 0.45–0.35 Ma [65, 66]. It seems that LCTs persist into the Late Acheulean with less variation in shape (often on large flakes) and with an increase in shaping refinement than the flaking methods and modalities permit [17]. Concepts of unity and diversity can be part of the same phenomena between Europe and Africa as part of similar and different ways to adapt to the local environment [28].

By the Late Acheulean, technological standardization and productive complexity that constitute major hallmarks of the LFA, were transformed into varied regional traditions. This phenomenon explains the many region‐specific denominations developed by prehistorians to describe or create a common language for the spatial distribution of cultural units with specific idiosyncrasies in Eurasia (e.g., the Clactonian, the Micoquien, the Tayacian, or even the biface‐less Early and Late Acheulean assemblages of western Europe [58, 67] and the Acheulo‐Yabrudian of the Levant). Advanced stone tool technologies provided advantageous solutions that gave hominins the capacity to dwell within relatively restricted geographic areas, as demonstrated by the advent of successive‐layer sites to which the hominins returned cyclically over long periods.

Another way to interpret the “Acheulean” that was broadly accepted during the conference, is as a category applied to an assemblage. Category means the definition is binary: an assemblage is either Acheulean or it is not; it cannot be “almost Acheulean” or “half Acheulean” or “Acheulean‐like”. In this case, Acheulean is considered as a property of a lithic assemblage linked to sets of behaviors. While the label may be extended to an archaeological locality or an individual artifact, it is defined and can only be evaluated at the level of a contextually delineated lithic assemblage. The underlying, often implicit, assumption of this categorical designation is that it refers to a distinct lineage of cultural transmission that emerged from a single, relatively well‐defined chrono‐spatial origin and dispersed from it; either by physical dispersal of hominins carrying it or through the propagation of abstract knowledge within existing hominin populations occupying different regions at different times [14, 15, 28, 59, 68, 69]. This raises questions, however, of cases of convergent evolution, as argued occasionally for the handaxes in eastern Asia, where researchers have raised the question whether these stone toolkits can be considered representative of a “true” Acheulean or not [28, 29, 31, 70, 71, 72, 73].

Regardless of the inconsistencies, cultural entities are not monolithic expressions and, as we have seen, do not appear at the same time or in a uniform way across Eurasia and Africa. As a result, the Acheulean can be characterized by all of the different elements mentioned above, even when not all of them are necessarily present in every assemblage; thus, accounting for observed temporal and spatial variation. What is clear from our discussions during the Lateurope conference is that the Acheulean corresponds to different cultural manifestations examined in the archaeological record by different researchers. In short, the Acheulean cultural unit involved a complex series of technological traits and social achievements that reflect the cumulative nature of culture and that can be observed to have occurred in all or only some of its expressions in Africa and Eurasia over a very long period of time. In other words, our definition of the Acheulean should not be reduced to a simple presence/absence of handaxes in the lithic assemblages. This could simply be a reflection of geographic and/or temporal variability and quite possibly related to dispersal/diffusion, or even site functionality or raw material constraints. If we briefly review the comments on the Acheulean from researchers working in various regions of Eurasia and Africa this should be quite evident. We begin with the African record.

## 4 Geographic Variability in How We Define the Acheulean

### 4.1 The African Record

Although early definitions of the Acheulean were originally based on western European assemblages, the African record provides the earliest and most abundant evidence for this technocomplex, positioning it as an important area of study where several hominin biological and cultural evolutionary patterns can be observed and reliably dated. Historically, the concept of the Acheulean has evolved from typological classifications largely based on the presence of handaxes, to a technocomplex characterized by specific cognitive, technological, and behavioral traits [45, 74, 75, 76]. The importance of this topic is underlined by the recent publication of several contributions in [77] focusing on how to perceive bifaces in the Palaeolithic archaeological record (e.g., see ref. [31]).

We argue that the Acheulean is best understood as a broad technocomplex of technological behaviors that distinguish it from the preceding Oldowan [78, 79] which marks a cognitive leap [46, 49, 79]. The hallmarks of the Acheulean technocomplex can be framed as a package of techno‐social behaviors that rarely or never occurred in Oldowan contexts [73]. These include the ability to produce large flakes and further shaping them into preconceived standardized tools [50], fragmented reduction sequences [43], and the hierarchical organization of knapping [78, 79, 80, 81], among others. LCTs (no matter their relative frequency) could be the best examples demonstrating the existence of mental templates and the ability to manage complex technological imperatives [44, 48]. Nonetheless, while LCTs are the most visible manifestation of these behaviors, they are not the sole factor indicating an Acheulean attribution [44, 60, 79]. Studies of the Acheulean in Africa, but also in Europe, reveal that this technology is characterized not only by the presence of LCTs but also by sophisticated small debitage flaking methods [60, 61, 78, 82], which represent cognitive advancements absent in earlier Oldowan industries.

### 4.2 The Asian Record

The Acheulean is irregularly distributed across Asia, where in some regions it appears in relatively high densities, while in others it is completely absent [13, 82]. Currently, the earliest evidence of the Acheulean in Asia is from the ‘Ubeidiya site (1.9–1.2 Ma) in Israel, and Attirampakkam (1.7–1.07 Ma, mean estimated average 1.5 Ma), Isampur (1.2 Ma), and other sites in western India (> 780 ka) [5, 6, 83, 84]. By the advent of the Chibanian, handaxe sites begin to appear more regularly in wider areas across Asia. For example, GBY in the southern Levant, dates to 0.78 Ma [85], and the sites from the Bose Basin in southern China [12, 29, 30, 31, 40, 86, 87] and the Go Da and Roc Tung sites in Vietnam [88] all date to the Early‐Middle Pleistocene transition by their association with Australasian tektites. It should be noted that there are many regions where handaxe sites, despite being widespread, are poorly dated; many Chibanian and Late Pleistocene sites in India are a case in point. By the beginning of the late Chibanian, handaxe “regions” appear, such as the Luonan Basin and Danjiangkou Reservoir Region in Central China [89, 90, 91, 92, 93] and the Korean sites in the Imjin/Hantan River Basins that are best exemplified by Chongokni (Jeongokni) [12, 70, 72, 94, 95]. Concurrently, in western Asia, the frequency of Late Acheulean assemblages dated to *ca*. 500 ka and younger increases significantly in the Arabian Peninsula, the Levant, and India [96, 97]. Comparative study of these different handaxe collections reinforces the understanding that there is a great deal of diversity present, with both variability and conservatism appearing, as attested to by the stone toolkits from such stratified Acheulean sequences as found at GBY [21, 82]. For instance, artifacts from Island Southeast Asia, all dating to the Chibanian or Late Pleistocene, are generally limited to small flakes that sometimes bear evidence of retouch. Heavy‐duty tools like handaxes, picks, polyhedrons and bolas have been recovered from the islands of Sumatra and Java in Indonesia and Luzon Island in the Philippines, but only on the surface [98, 99, 100, 101, 102, 103].

For decades, discussions about the Acheulean in Asia have focused largely on the validity of the “Movius Line”, which purportedly served as a geographical and cultural boundary, separating the Acheulean traditions of the West from the supposedly “conservative” Mode 1 industries of eastern Asia [13]. The handaxe assemblages in eastern Asia have not often been considered “true” Acheulean due to low frequencies of LCTs, perceived morphological “crudeness”, and their relatively late chronological appearance [30, 31, 33, 70, 91, 93, 104, 105, 106]. In fact, some scholars have suggested that the eastern Asian LCTs represent instances of convergent evolution rather than a shared cultural tradition with western Eurasia and Africa by cultural transmission [29, 30, 71, 72, 73]. Other scholars hold a different view, arguing that the Acheulean technology in eastern Asia exhibits noticeable regional diversity and variation, which cannot be adequately captured by uniform or simplistic characterizations as originally proposed by Movius [104, 107]. For instance, LCTs in eastern Asia display considerable diversity and variability in raw material selection, blank types, shaping strategies, and morphological features [29, 92]. Moreover, it may be argued that Acheulean sites in eastern Asia demonstrate broad spatial distribution and temporal continuity, while the earliest appearance of Acheulean technology in southern China's Bose Basin could actually be evidence of cross‑regional technological transmission and diffusion [31, 104, 108]. These competing models serve to raise a key question: what defines a site as “Acheulean” in eastern Asia?

The relative paucity of large concentrations of typical Acheulean handaxes in eastern Asia may actually create new paths to redefine an Acheulean technological “package” even in the absence of LCTs, which can be applied specifically for understanding the eastern Asian record (and even areas like Central Europe that have no evidence of the Acheulean). For instance, the archaeological record of the Nihewan Basin in northern China has traditionally been attributed to Mode 1 (Oldowan‐like) technology due to the lack of LCTs. However, recent analyses challenge this view. Assemblages such as Xiaochangliang (1.36 Ma) [105], Madigou (1.2 Ma) [109], Feiliang (1.2 Ma) [110], and Donggutuo (1.1 Ma) [106, 111] display varying degrees of small retouched tools distinct from the typical African Oldowan. Furthermore, recent findings by Ma et al. [112] at Cenjiawan (1.1 Ma) reveal prepared core technology and standardized reduction sequences. This evidence suggests technological abilities that may parallel African Mode 2 hominins without LCTs [112]. Such evidence as found in the Nihewan Basin does raise the idea of rethinking how the Acheulean or broader cultural sequences may be identified in eastern Asia, a point raised more than two decades ago by Gao and Norton [7].

### 4.3 The European Record

The current archaeological and chronological data record a late occupation of western Europe at around 1 Ma during the Early‐Middle Pleistocene Transition (EMPT) that may be due to hominins coming from Asia, likely over a period of time in waves, a region that now has evidence of occupation dating back close to the beginning of the Early Pleistocene [12, 113, 114, 115, 116]. Then, over time, a limited number of sites in Europe yield early evidence of LCTs, including bifaces. These sites (Barranc de la Boella in Spain, Moulin Quignon, la Caune de l'Arago (Q and P levels), and la Noira in France, Notarchirico in Italy, and Fordwich and Brandon Fields in England), all dated between 900 and 650 ka, are located in northwestern and southern Europe [23, 26, 60, 61, 66, 117, 118, 119, 120, 121]. These new strategies only appeared west of the Rhine Valley, which is considered to have served as a geographical barrier as there are no handaxe assemblages so far reported from central Europe. While these assemblages may be tentatively assigned to the “Early Acheulean” they do in fact appear relatively late compared to East Africa (1.9–1.5 Ma), the Levant (Ubeidiya at 1.9–1.2 Ma) and broader Asia (1.5–1.2 Ma). As such, this European evidence opens the possibility to discuss the introduction of new traditions between MIS 18 and 12.

The Spanish site of Barranc de la Boella is the oldest occurrence in western Europe that has produced evidence of crudely made LCTs, including picks and cleavers, but (so far) no bifaces [23]. Between Barranc de la Boella, dated to around 900 ka, and sites dated to 700 ka (Notarchirico, Italy; La Noira stratum A and Moulin Quignon, France), there is a chronological gap with no clear and well‐dated evidence of local development of biface production [122]. The Q and P levels at the French site of La Caune de l'Arago (ca. 550 ka) have yielded a rich series of assemblages characterized by symmetrical bifaces, but no cleavers [123]. This raises questions regarding whether the repeated appearance of this technocomplex should be seen as punctuated events that coincide with the arrival of new hominin groups in western Europe. Given the complexity of identifying taxonomically exactly who these hominins were (see discussion above), it is usually premature to try to assign a single species of *Homo* to these lithic toolkits (refer to the Notarchirico example discussed above). However, the level TD6.2 at the Atapuerca site (Spain) presents a clear core‐and‐flake industry that dates to 900 ka that has been associated specifically with *H. antecessor* [63]. The TD6.2 materials include some flakes exceeding 10 cm and evidence of some complexity in the core technology with the presence of some bifacial centripetal cores [63, 124]. At this time, for western Europe between 900 ka to 700 ka, the archaeological record suggests some degree of continuity in core technology with occasional appearances of more complex stone tools [17].

From 700 to 500 ka, before MIS 12, bifaces still do not appear uniformly in western Europe. Examples of biface sites from this time period include Brandon Fields (MIS 15 [119]) and Boxgrove (MIS 13, Roberts et al. 1999) in the United Kingdom (UK), reflecting a shift from more variable and expedient shaping towards a highly standardized technological system [17], or High Lodge (Bed C) in the UK, with an elaborate scraper production that appeared during MIS 13 [119]. Biface technology persists until 180 ka in some areas, as in the Northwest Iberian Peninsula and Central Spain [16, 125]. Once again, this raises questions about how to define the European “Acheulean” and Lower Palaeolithic. For some, innovations in core technologies that appear widely across the region, such as bifacial and unifacial centripetal core management and overcoming raw material constraints, suggest a common background of traditions and technological packages in western Europe around 700 ka, with or without LCTs. Similarly, others suggest a complex of localized lithic expressions, with or without LCTs, but a foundation of technical knowledge shown through improved hunting, butchery, bone‐ and wood‐working (e.g., see refs. [126]).

To summarize the early evidence of assemblages with bifaces in the European record (700–550 ka):

1. The predominant use of local (within 5 km of the site) raw materials with only occasional evidence of stone having been moved longer distances, such as Caune de l'Arago, France [123], or Waverley Wood, UK, as well as tool types produced on certain types of raw materials. However, it has been observed that sometimes when higher quality raw materials were present, they were preferentially used over other poorer quality stones that were also available (cf [127]).
2. Occasional complexity (e.g., centripetal core management) in large and small flake production, sometimes depending on the size of the available stone.
3. Adaptation to raw material constraints suggesting flexibility and some evidence of flaking independently of stone geometry.
4. Relatively few bifaces when present.
5. Sometimes a large diversity of crudely‐made LCTs.
6. Rare use of large flakes (mainly Iberian Peninsula) for shaping LCTs.
7. Diversity of shaping modes and forms for bifaces (form of standardization) and bifacial tools (non‐standardization) with some evidence of soft‐hammer percussion.
8. Absence of cleavers on flakes in the Northwest, but presence of some cleaver‐like tools.
9. Various light‐duty tools, both on flakes or natural stones with one or several retouched cutting edges, convergent edges, pointed extremities and denticulation/notches.

## 5 Out of Africa I Revisited

One question that was briefly touched upon during the Lateurope conference concerns the delayed arrival of hominins in Europe relative to Asia, an issue that can be considered within the framework of the “Out of Africa I” dispersal model. Briefly, this model posits that hominins evolved only in Africa and then moved out of Africa and into Eurasia sometime after the middle Early Pleistocene (between 1.8 Ma and 1.0 Ma). The hominin that is often considered to be the early disperser is *Homo erectus sensu lato*, which roughly coincides with the appearance of the Acheulean technology in Africa. The exact timing of this dispersal and whether the only hominin to have made these early movements was *H. erectus* is now being questioned however, particularly given the growing number of sites across Asia that date to the early Early Pleistocene and the understanding that a great deal of morphological overlap existed between early *Homo* (*H. habilis*, *H. rudolfensis*) and *H. erectus/ergaster* [12, 36, 87, 113, 115, 116, 128, 129, 130, 131]. In other words, it seems likely that either the origin of *H. ergaster*/*erectus* needs to be pushed back to the Plio‐Pleistocene transition or we need to explore whether other hominins (e.g., *H. habilis*/*rudolfensis*, australopiths) were actually the first to move out of Africa [12, 115, 131]. Given that the Lomekwian appears in the same geological age as *Kenyanthropus platyops* and *A. afarensis*, and *Paranthropus* was recently suggested to have used stone tools [132], it may be possible we have been underestimating the behavioral capabilities of some of these older hominin groups. It would now appear that well before hominins (e.g., WT 15000 “Nariokotome Boy”) fell within the same body size range as modern humans they were moving out of Africa.

Further, maps that outline possible hominin movements across Eurasia need to be reconsidered in light of the new data becoming available. For instance, the “big arrow” coming out of Africa and splitting into 2 directions (east and west) appears to be a bit of an oversimplification, as are ideas about purposeful directional movements. A case in point may be the unidirectional models that show hominins only moved East to West across Europe. Although these points were only touched on during the Lateurope conference, they will be further investigated in the future.

## 6 Moving Forward

The strongest defendable stance is therefore that the Acheulean is a valid and useful high‐level analytical category provided it is explicitly treated as a techno‐complex and not reified into a culturally unitary entity. This is a point that was widely agreed upon during the conference. This status is not a weakness but an epistemic specification: the Acheulean is real at the level of technological practice families, while its finer‐grained cultural interpretations require regionally and chronologically bounded demonstrations rather than definitional assumptions (**Acheulean s.l./s.s**.). There was a broad agreement that future research should prioritize the reconstruction of variability and diachronic change in different regions of Eurasia and Africa. In Africa, more effort should be paid to study the small debitage. In Asia, emphasis should be placed on further excavations, obtaining dates from regions with rich records (e.g., India) and focusing on technological features that reflect the cognitive abilities of toolmakers—such as mental templates and hierarchical planning—rather than relying solely on the typology of the final products. Even after the conference, points of disagreement remained. For instance, how are Acheulean sites defined in China and what is the meaning and relevance of the Movius line concept. As a categorical concept that is, in essence, dichotomous, the boundaries of the Acheulean can only be defined in an unambiguous manner with respect to a clear metric of lithic technology at the assemblage level.

While there are some factors that serve to link the diverse expressions of the Acheulean in different regions into a more or less coherent stage of techno‐social evolution, we conclude here that it must not be considered as a single phenomenon. Moving beyond typological definitions is a key question to define the Acheulean across different geographic areas, with the role and diagnostic value of the LCTs considered as one feature among others (e.g., form variability, shaping intensity, etc.). Often simplified into the simple presence/absence of bifaces or LCTs *sensu stricto*, in reality, the Acheulean must be considered in light of a far wider and more complex series of milestones that mark the evolution of human culture broadly speaking. The results of the Lateurope conference is one step in the right direction toward developing this deeper understanding of the Acheulean.

## Data Availability

Data are available under request to the two corresponding authors.
